# Mitochondrial Damage-Associated Molecular Patterns of Injured Axons Induce Outgrowth of Schwann Cell Processes

**DOI:** 10.3389/fncel.2018.00457

**Published:** 2018-11-27

**Authors:** Andrea Korimová, Ilona Klusáková, Ivana Hradilová-Svíženská, Marcela Kohoutková, Marek Joukal, Petr Dubový

**Affiliations:** Department of Anatomy, Division of Neuroanatomy, Faculty of Medicine, Masaryk University, Brno, Czechia

**Keywords:** RT4-D6P2T schwannoma cells, *in vitro*, fMLP, CpG ODN, FPR2, TLR9, growth conus, nerve injury

## Abstract

Activated Schwann cells put out cytoplasmic processes that play a significant role in cell migration and axon regeneration. Following nerve injury, axonal mitochondria release mitochondrial damage-associated molecular patterns (mtDAMPs), including formylated peptides and mitochondrial DNA (mtDNA). We hypothesize that mtDAMPs released from disintegrated axonal mitochondria may stimulate Schwann cells to put out cytoplasmic processes. We investigated RT4-D6P2T schwannoma cells (RT4) *in vitro* treated with N-formyl-L-methionyl-L-leucyl-phenylalanine (fMLP) or cytosine-phospho-guanine oligodeoxynucleotide (CpG ODN) for 1, 6 and 24 h. We also used immunohistochemical detection to monitor the expression of formylpeptide receptor 2 (FPR2) and toll-like receptor 9 (TLR9), the canonical receptors for formylated peptides and mtDNA, in RT4 cells and Schwann cells distal to nerve injury. RT4 cells treated with fMLP put out a significantly higher number of cytoplasmic processes compared to control cells. Preincubation with PBP10, a selective inhibitor of FPR2 resulted in a significant reduction of cytoplasmic process outgrowth. A significantly higher number of cytoplasmic processes was also found after treatment with CpG ODN compared to control cells. Pretreatment with inhibitory ODN (INH ODN) resulted in a reduced number of cytoplasmic processes after subsequent treatment with CpG ODN only at 6 h, but 1 and 24 h treatment with CpG ODN demonstrated an additive effect of INH ODN on the development of cytoplasmic processes. Immunohistochemistry and western blot detected increased levels of tyrosine-phosphorylated paxillin in RT4 cells associated with cytoplasmic process outgrowth after fMLP or CpG ODN treatment. We found increased immunofluorescence of FPR2 and TLR9 in RT4 cells treated with fMLP or CpG ODN as well as in activated Schwann cells distal to the nerve injury. In addition, activated Schwann cells displayed FPR2 and TLR9 immunostaining close to GAP43-immunopositive regenerated axons and their growth cones after nerve crush. Increased FPR2 and TLR9 immunoreaction was associated with activation of p38 and NFkB, respectively. Surprisingly, the growth cones displayed also FPR2 and TLR9 immunostaining. These results present the first evidence that potential mtDAMPs may play a key role in the induction of Schwann cell processes. This reaction of Schwann cells can be mediated via FPR2 and TLR9 that are canonical receptors for formylated peptides and mtDNA. The possible role for FPR2 and TLR9 in growth cones is also discussed.

## Introduction

Wallerian degeneration (WD) is the highly orchestrated cascade of cellular and molecular events distal to injury of nerve fibers, and is considered to be a kind of innate immune reaction or sterile inflammation (Stoll et al., 2002; Gaudet et al., 2011). These events comprise, among others, invasion of macrophages and other immune cells, axon fragmentation, as well as activation and inflammatory profiling of Schwann cells (Stoll et al., 2002; Dubový et al., 2013). Schwann cells of the injured nerve and the so-called terminal Schwann cells overlying denervated neuromuscular junctions elaborate processes that guide the sprouts of regenerated axons (Son and Thompson, 1995a,b; Gomez-Sanchez et al., 2017). Thus, cytoplasmic extensions of Schwann cells are crucial for axon regeneration in the peripheral nervous system. However, the stimuli that trigger the elaboration of Schwann cells processes have still not been precisely characterized.

Degeneration of a distal nerve stump is the source of a broad spectrum of damage-associated molecular patterns (DAMPs) produced by the digestion of the endoneurial extracellular matrix as well as fragmentation of axons and their myelin sheaths (Karanth et al., 2006; Boivin et al., 2007; Kato and Svensson, 2015). These DAMPs are ligands for pattern recognition receptors such as the toll-like receptors (TLRs) that regulate WD and Schwann cell inflammatory profiling (Boivin et al., 2007; Goethals et al., 2010; Boerboom et al., 2016; Dubový, 2017).

The axons and their terminals contain abundant mitochondria which are disintegrated distal to a peripheral nerve lesion. The disintegrated mitochondria release specific mitochondrial DNA (mtDNA) fragments and proteins like formyl peptides generally termed mitochondrial damage-associated molecular patterns (mtDAMPs; Krysko et al., 2011). Formylated peptides are ligands for the formylpeptide receptor 2 (FPR2; Le et al., 2002) while mtDNA activates the toll-like receptor 9 (TLR9; Zhang et al., 2010).

We hypothesize that mtDAMPs of disintegrated axonal mitochondria in the immediate aftermath of WD can stimulate Schwann cells to put out cytoplasmic processes through the action of the corresponding receptors. In the experiments presented here, we used the rat RT4-D6P2T schwannoma cell line as a model for studying the elaboration of cytoplasmic processes induced by the potential mtDAMPs, formyl-methionyl-leucyl-phenylalanine (fMLP) and cytosine-phospho-guanine oligodeoxynucleotide (CpG ODN) known to be the prototypical ligands of FPR2 and TLR9, respectively (Le et al., 2002; Chen et al., 2011). In addition, we detected FPR2 and TLR9 in activated Schwann cells distal to the nerve injury but close to regenerated axons.

## Materials and Methods

### Cell Culture and Treatment

Rat RT4-D6P2T schwannoma cell line (RT4) was provided by ATCC. RT4 cells were cultivated in Dulbecco’s Modified Eagle’s Medium/Nutrient F-12 Ham (DMEM/F12) at 37°C in a 5% CO_2_ atmosphere. The medium was supplemented with 10% Fetal Bovine Serum (FBS), 2 mM L-glutamine and antibiotics (100 U/ml penicillin and 100 μg/ml streptomycin; all obtained from Sigma Aldrich). At approximately 90% confluency, cells were seeded at a density of 1 × 10^4^ cells/cm^2^ and harvested in serum-free medium before treatment.

To determine changes in cytoplasmic process extension, RT4 cells were stimulated with the appropriate mtDAMPs. fMLP (Sigma-Aldrich) dissolved in dimethyl sulfoxide (DMSO) as a 100 mM stock solution and maintained at −20°C was used in 100 nM, 10 μM and 50 μM concentration for 1, 6 and 24 h and equivalent volumes of DMSO (0.02%, 0.2% and 1%) were used in controls. To test if the fMLP effect is mediated by FPR2, a set of cells was preincubated with 1 μM PBP10 (Tocris), a selective inhibitor of FPR2 (Forsman et al., 2012), for 20 min before treatment with 50 μM fMLP.

RT4 cells were treated for the same durations, with immunostimulatory 1 μM CpG ODN (ODN D-SL03, a C class CpG ODN, 5′-tcg cgaacgttcgccgcgttcgaacgcgg-3′, Invivogen) directly or following a pretreatment with 1- or 10-fold amounts of inhibitory ODN (INH ODN) (1 or 10 μM, ODN 4084-F, 5′-cctggatgggaa-3′, Invivogen) for 30 min. Oligonucleotides tested for absence of bacterial contamination were separately resuspended in endotoxin-free water to 500 μM stock solution according to the manufacturer’s instructions, aliquoted and stored at −20°C until used.

### Immunofluorescent Staining and Quantitative Analysis of Cytoplasmic Processes

At the end of the treatments described, cells cultured on slides in 12-well plates were fixed with 4% paraformaldehyde in phosphate-buffered saline (PBS) for 5 min, washed three time with PBS, and permeabilized with cold methanol:acetone (1:1). The cells were then immunostained with rabbit monoclonal anti-β-actin (1:200; Cell Signaling) overnight and FITC-conjugated donkey anti-rabbit affinity-purified secondary antibody (1:100; Millipore) for 90 min at room temperature. The slides with cells were washed extensively in PBS and mounted with the anti-fading medium Mowiol.

To detect FPR2, TLR9 or paxillin expression, portions of the RT4 cells were immunostained overnight with rabbit polyclonal anti-FPR2 (1:100; Novusbio), anti-TLR9 antibody (1:500; Acris) or a mouse monoclonal antibody recognizing tyrosine-phosphorylated paxillin (1:1,000; Chemicon). The immunohistochemical reaction was visualized using TRITC-conjugated donkey anti-rabbit or anti-mouse affinity-purified secondary antibodies (1:100; Millipore) for 90 min at room temperature. Control cells for immunohistochemical staining were incubated without primary antibody or by substituting the primary antibodies with the donkey IgG isotype. Cell nuclei were stained with Hoechst 33342, and the slides were mounted in aqueous mounting medium (Vectashield; Vector Laboratories, Burlingame, CA, USA). The stained RT4 cells were analyzed, and pictures captured using an epifluorescence microscope (Nikon Eclipse NI-E Motorized Microscope System) equipped with a Nikon DS-Ri1 camera (Nikon, Prague, Czechia).

Actin immunostained RT4 cells were used for quantitative analysis of cytoplasmic processes in response to stimuli. At least 50 cells were randomly chosen for each experimental group and the number of cytoplasmic processes per cell was manually counted using the Count and Taxonomy module of NIS- Elements software (Nikon, Prague, Czechia) following curve fitting detection of cell body boundaries. The number of cytoplasmic processes was measured by a person blind to the experimental conditions.

Dimethyl sulfoxide was used as a solvent and vehicle for fMLP. Because it was demonstrated that medium supplemented with DMSO was enough to induce changes in cell surface area (Lemieux et al., 2011), we compared number of cell processes after fMLP treatment to those of cells cultivated in medium supplemented with DMSO of a corresponding concentration used for dissolution of fMLP and for the appropriate durations.

### Western Blot Analysis

As activated paxillin is involved in the initiation of cytoplasmic processes outgrowth in the cell (López-Colomé et al., 2017), we quantified the level of paxillin phosphorylated in tyrosine positions by western blot analysis in RT4 cells after potential mtDAMP action. After cultivation in the presence or absence of stimulants or inhibitors, RT4 cells were washed twice with ice-cold PBS, mechanically harvested from the culture dishes and sonicated for 30 s. The samples were centrifuged at 2,000 rpm for 5 min and lysed at 4°C in buffer containing 80 mM HEPES, pH 7.5; 2.5 M urea, 1 mM EDTA, 0.5% Triton X-100 and 20 mM β-mercaptoethanol as well as cocktails of protease and phosphatase inhibitors (Roche, Germany). Equal amounts of proteins from cell lysates (50 μg/lane) were separated by SDS-polyacrylamide gel electrophoresis and transferred to a nitrocellulose membrane (Bio-Rad). After blocking of nonspecific binding sites with 5% bovine serum albumin (BSA) in TRIS-buffered saline (pH 7.2) for 2 h, membranes were incubated with mouse monoclonal anti-tyrosine-phosphorylated paxillin (1:1,000; Chemicon) at 4°C overnight, followed by peroxidase-conjugated anti-mouse IgG (1:1,000; Sigma, Ronkonkoma, NY, USA) at room temperature for 1 h. Protein bands were visualized using the ECL detection kit (Bio-Rad) on a PXi chemiluminometer reader and analyzed using the GeneTools densitometry software (Syngene). All analyzed proteins were normalized to β-actin.

### Animals and Surgical Treatment

The *in vivo* experiments were performed in 15 adult male rats (Wistar, 250–280 g, Anlab, Brno, Czechia) housed on 12 h light/dark cycles at a temperature of 22–24°C under specific pathogen-free conditions in the animal housing facility of Masaryk University. Sterilized standard rodent food and water were available *ad libitum*. Animals for surgical treatments were anesthetized using a mixture of ketamine (40 mg/ml) and xylazine (4 mg/ml) administered intraperitoneally (0.2 ml/100 g body weight). All surgical procedures were carried out under sterile conditions by the same person according to protocols approved by the Animal Investigation Committee of the Faculty of Medicine, Brno, Czechia.

The right ulnar nerve of three rats was exposed in mid-thigh, ligated with two ligatures and cut. The proximal nerve stump was buried and fixed in muscles to protect the distal stump from reinnervation. The right ulnar nerve of three other rats was exposed for a short segment and crushed with clamp of a defined force of 1.9 N twice for 1 min (Ronchi et al., 2010) under a stereological microscope. The distal margin of the crush injury was indicated with Indian ink and the skin wound was closed with 5/0 sutures. The ulnar nerve of sham-operated rats (*n* = 3) was carefully exposed without any lesion. To demonstrate a role of p38 and NFkB in downstream signaling pathways of FPR2 and TLR9, the right ulnar nerve of four rats was crushed as described above and 10 μl of PBP10 (1 μM; Tocris) or chloroquine (50 μM; InvivoGen) was injected via a micro syringe into the subarachnoid space of the cisterna magna (Dubový et al., 2018). The inhibitor of FPR2 (PBP10) or TLR9 (chloroquine) was dissolved in artificial cerebrospinal fluid (ACSF; Hylden and Wilcox, 1980). Ten microliter of ACSF was injected in two control rats. All operated rats were left to survive for 3 days.

### Immunofluorescence Staining of Activated Schwann Cells Distal to Nerve Injury

After the period of survival, the animals were deeply anesthetized with a lethal dose of sodium pentobarbital (70 mg/kg body weight, i.p.) and perfused transcardially with 500 ml PBS (10 mM sodium phosphate buffer, pH 7.4, containing 0.15 M NaCl) followed by 500 ml of Zamboni’s fixative (Zamboni and Demartin, 1967). The right ulnar nerves of sham-operated rats, distal stumps of transected and crushed ulnar nerves were removed and immersed in Zamboni’s fixative overnight. After washing with 10% sucrose in PBS, longitudinal cryostat sections of 10 μm thickness were cut.

The sections were washed with PBS containing 0.05% Tween 20 (PBS-T) and 1% BSA for 10 min, treated with 5% normal donkey serum in PBS-T for 30 min and immunostained. The longitudinal sections prepared from nerve segments of sham-operated animals and nerve segments distal to nerve transection were incubated under the same conditions with rabbit polyclonal anti-FPR2 (1:100; Novusbio) or anti-TLR9 (1:500; Acris) primary antibodies and TRITC-conjugated and affinity-purified donkey anti-rabbit secondary antibody (1:100; Millipore). One portion of the sections was double immunostained for GFAP and FPR2 or TLR9 to detect these receptor proteins in activated Schwann cells. Briefly, the sections were incubated with rabbit polyclonal anti-FPR2 or anti-TLR9 antibodies and then with chicken polyclonal anti-GFAP antibody (1:500; Abcam), in each primary antibody overnight. To visualize the immunoreaction, the sections were incubated with TRITC-conjugated and affinity-purified donkey anti-rabbit secondary antibody, while FITC-conjugated donkey anti-chicken secondary antibody (both 1:100; Millipore) was used for development of GFAP immunostaining. Control sections were incubated without primary antibodies as well as with rabbit or chicken polyclonal antibodies and treated with FITC-conjugated donkey anti-chicken or TRITC-conjugated donkey anti-rabbit secondary antibodies, respectively. No immunofluorescence staining was observed in the control sections (data not shown).

To visualize activated Schwann cells close to growing axons, the longitudinal sections distal to ulnar nerve crush were double immunostained with mouse monoclonal anti-GAP43 (1:500; Sigma) and chicken polyclonal anti-GFAP antibodies. The immunostaining was visualized with FITC-conjugated donkey anti-mouse and TRITC-conjugated donkey anti-chicken secondary antibodies.

For evidence of FPR2 or TLR9 immunopositivity in cells close to growing axons, the sections were incubated with mouse monoclonal anti-GAP43 (1:500; Sigma) and rabbit polyclonal anti-FPR2 (1:100; Novusbio) or anti-TLR9 (1:500; Acris) antibodies. Moreover, double immunostaining with rabbit polyclonal anti-FPR2 and mouse monoclonal anti-phosphorylated p38 MAPK (1:100; Santa Cruz) antibodies as well as mouse monoclonal anti-TLR9 (1:100; Novusbio) and rabbit monoclonal anti-pNFkB(p65) (1:100; Cell Signaling) antibodies was used to determine downstream signaling pathways of FPR2 and TLR9. The nerve sections of ACSF- and PGP10- or chloroquine-treated rats were double immunostained under the same conditions. Double immunofluorescence was developed with corresponding FITC- and TRITC-conjugated affinity-purified donkey anti-mouse and anti-rabbit secondary antibodies (1:100; Millipore). Control sections were incubated without primary antibody.

All sections were stained with Hoechst 33342 to detect cell nuclei, mounted in aqueous mounting medium (Vectashield; Vector Laboratories, Burlingame, CA, USA) and analyzed using an epifluorescence microscope (Nikon Eclipse NI-E Motorized Microscope System) equipped with a Nikon DS-Ri1 camera (Nikon, Prague, Czechia).

## Results

### Quantitative Analysis of Cytoplasmic Processes in RT4 Cells Induced by fMLP or CpG

**Figure 1:**
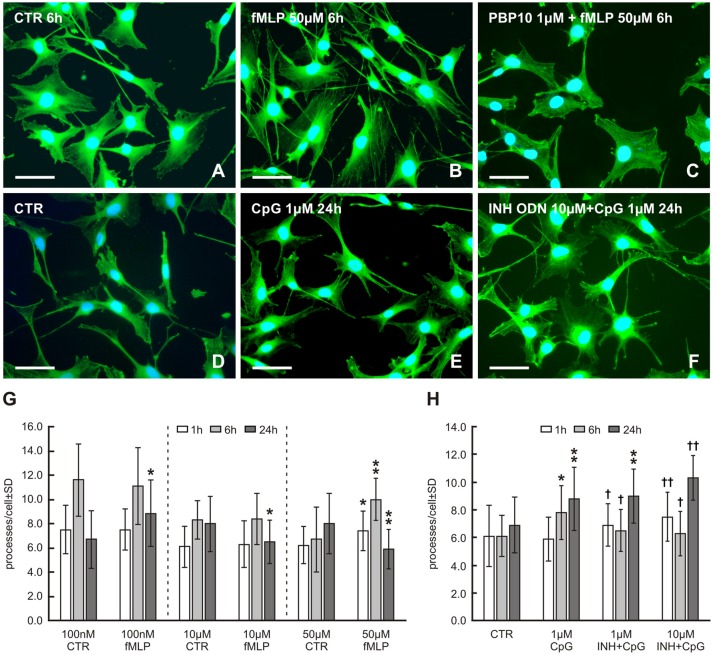
Representative pictures of RT4-D6P2T cells cultivated in control medium **(A,D)** and medium with 50 μM formyl-L-methionyl-L-leucyl-phenylalanine (fMLP) **(B)** for 6 h or 1 μM cytosine-phospho-guanine oligodeoxynucleotide (CpG ODN) **(E)** for 24 h showing an increased number of cytoplasmic processes. Preincubation of RT4 cells with the formylpeptide receptor 2 (FPR2) inhibitor PBP10 (1 μM) resulted in a noticeable reduction of cytoplasmic processes in comparison to only fMLP treatment **(C)**. An additive effect of 10 μM of inhibitory ODN (INH ODN) pre-treatment for 30 min before 1 μM CpG ODN for 24 h on the development of cytoplasmic processes is shown in **(F)**. After *in vitro* cultivation and treatment, the cells were fixed and immunostained with rabbit monoclonal anti-β-actin antibody and FITC-conjugated donkey anti-rabbit secondary antibody. Cell nuclei were stained with Hoechst 33342. Scale bars = 50 μm. **(G)** The graph illustrates mean number of cytoplasmic processes per cell of the RT4-D6P2T cell line cultivated for different time periods (1, 6 and 24 h) with fMLP at 100 nM, 10 μM and 50 μM concentrations and controls (CTR) containing the appropriate volume of dissolved in dimethyl sulfoxide (DMSO). *p < 0.05 when compared with DMSO control, **p < 0.001 when compared with DMSO control. **(H)** The graph illustrates mean number of cytoplasmic processes per cell of the RT4-D6P2T cell line cultivated for different time periods (1, 6 and 24 h) with control medium (CTR), 1 μM CpG ODN (CpG) and cells pre-treated with 1 μM or 10 μM of INH ODN (INH) for 30 min before treating with CpG ODN. *p < 0.05 compared with control, **p < 0.001 compared with control, ^†^*p* < 0.05 INH ODN pre-treatment compared with CpG ODN, ^††^*p* < 0.001 INH ODN pre-treatment compared with CpG ODN.

To investigate whether the potential mtDAMPs fMLP or CpG can induce outgrowth of Schwann cells processes, RT4 cells were cultured *in vitro* with 100 nM, 10 μM and 50 μM concentrations of fMLP or 1 μM CpG ODN for 1, 6 and 24 h. Representative pictures illustrating changes of cytoplasmic processes in RT4 cells after treatment with fMLP and CpG in comparison to controls as well as following pretreatment with the FPR2 inhibitor PBP10 or INH ODN are shown in Figure 1.

Because fMLP was dissolved in DMSO, it was necessary to compare values of cytoplasmic processes measured in RT4 cells after fMLP treatment to control values obtained in RT4 cells cultivated with the corresponding concentration of DMSO used to dissolve fMLP. fMLP influenced the formation of RT4 cell processes very differently under different concentrations and treatment durations (Figure 1G). No effect on the number of cell processes was demonstrated in low concentrations of fMLP (100 nM and 10 μM) after 1 h and 6 h, whereas a higher concentration of fMLP (50 μM) for the same duration resulted in a significant increase in the number of cytoplasmic processes per cell (1 h: 6.2 ± 1.7 vs. 7.4 ± 1.8, *p* < 0.05; 6 h: 6.7 ± 2.7 vs. 10.0 ± 2.2, *p* < 0.001). Nevertheless, after 24 h, 100 nM fMLP resulted in a significantly increased number of cytoplasmic processes per cell (6.7 ± 2.4 vs. 8.4 ± 2.6, *p* < 0.05), but higher concentrations (10 μM and 50 μM) reduced the number of the processes when compared to controls (Figure 1G). Because the greatest increase in the number of cytoplasmic processes was induced by 50 μM fMLP treatment for 6 h, these conditions were used to test the effect of FPR2 inhibition. A 20 min preincubation with 1 μM PBP10 as an FPR2 inhibitor before 50 μM fMLP treatment resulted in a significant reduction of cytoplasmic process outgrowth when compared with the standard treatment for 6 h (6.9 ± 2.3 vs. 10.0 ± 2.2, *p* < 0.001; see also Figure 1C).

CpG ODN treatment for 6 and 24 h induced a significantly increased number of cytoplasmic processes compared to control cells (6 h: 7.8 ± 2.5 vs. 6.1 ± 1.5, *p* < 0.05; 8.8 ± 2.7 vs. 6.9 ± 2.0, *p* < 0.001). However, CpG ODN treatment for 1 h did not significantly affect the number of cytoplasmic processes (Figure 1H). A pre-treatment with 1 or 10 μM INH ODN before 1 or 24 h CpG ODN stimulation unexpectedly resulted in significantly increased outgrowth of cytoplasmic processes when compared to control cells or cells treated only with CpG ODN (Figures 1F,H). In contrast, pre-treatment with 1 or 10 μM INH ODN and subsequent stimulation with CpG ODN for 6 h caused the expected inhibitory effect and the mean number of cell processes was reduced when compared with only CpG ODN treatment (Figure 1H).

### Immunohistochemical Detection of FPR2, TLR9 and Analysis of Activated Paxillin in RT4 Cells

**Figure 2:**
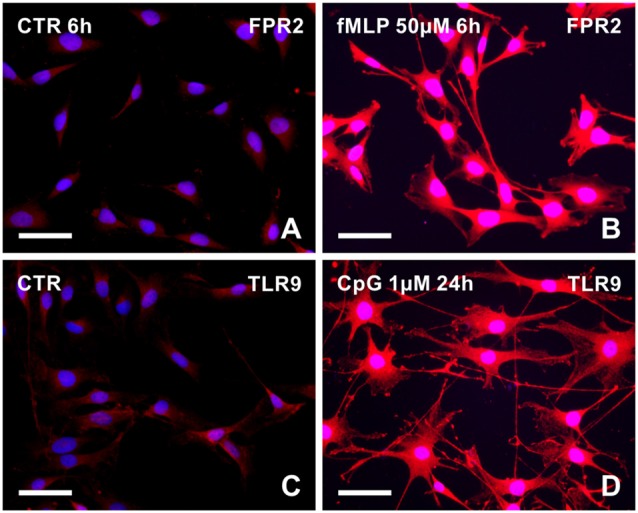
Representative pictures illustrating FPR2 or toll-like receptor 9 (TLR9) immunostaining in RT4-D6P2T cells cultivated in control medium (**A,C**, respectively) and increased immunofluorescence intensity after treatment with 50 μM fMLP for 6 h **(B)** or 1 μM CpG ODN for 24 h **(D)**. Both control and treated cells were immunostained under the same conditions with rabbit polyclonal anti-FPR2 or anti-TLR9 antibody and TRITC-conjugated donkey anti-rabbit secondary antibody. Cell nuclei were stained with Hoechst 33342. Scale bars = 50 μm.

To prove that fMLP and CpG can induce changes of cytoplasmic processes in RT4 cells via the corresponding receptors, we detected FPR2 and TLR9 expression in control and treated cells. RT4 cells cultured in medium with fMLP (50 μM) for 6 h displayed increased intensity of FPR2 immunofluorescence in contrast to control cells cultivated in medium only with the corresponding concentration of DMSO (Figures 2A,B). Similarly, untreated control RT4 cells displayed a weak TLR9 immunofluorescence whereas those cultivated in medium with CpG (1 μM) for 24 h showed a more intense immunofluorescence (Figures 2C,D).

**Figure 3:**
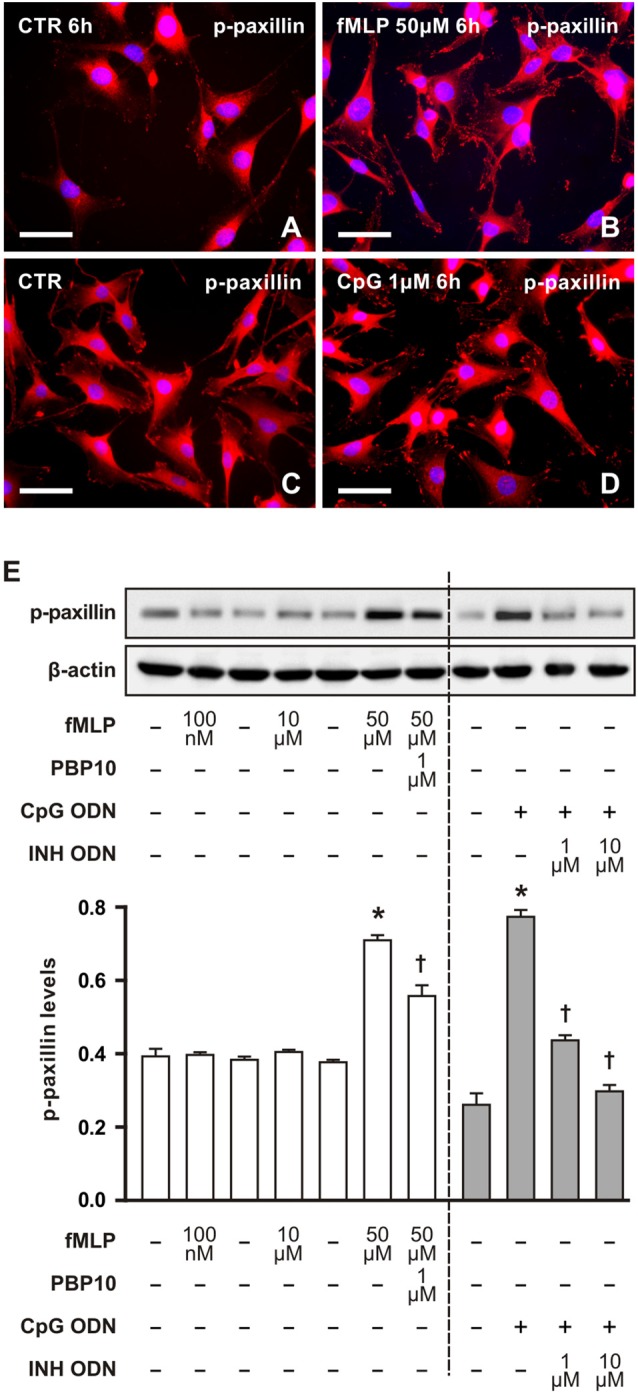
Representative pictures of RT4-D6P2T cells cultivated in control medium displayed low immunofluorescence intensity for phosphorylated paxillin **(A,C)** in comparison to increased immunofluorescence intensity after treatment with 50 μM fMLP **(B)** for 6 h or 1 μM CpG ODN for 6 h **(D)**. The cells were immunostained with mouse monoclonal antibody recognizing paxillin phosphorylated in tyrosine positions and TRITC-conjugated donkey anti-mouse secondary antibody. Cell nuclei were stained with Hoechst 33342. Scale bars = 50 μm. **(E)** Upper panel shows a representative western blot of phosphorylated paxillin (p-paxillin) protein analyzed in RT4-D6P2T cells cultivated in control medium (−) and the cells after treatment with fMLP (100 nM, 10 μM and 50 μM) for 6 h or with 1 μM PBP10 preincubation and treatment with 50 μM fMLP for 6 h. Right-sided set shows a representative western blot of phosphorylated paxillin (p-paxillin) protein in RT4-D6P2T cells cultivated in control medium (−) and after 1 μM CpG ODN (+) treatment for 6 h or pre-treatment with INH ODN (1 μM, 10 μM). The graph below illustrates mean values ± SD of p-paxillin protein density relative to values of untreated cells from three independent experiments. *p < 0.05 when compared to fMLP or CpG ODN stimulation control, ^†^*p* < 0.05 when compared to fMLP or CpG treatment.

We also detected phosphorylated paxillin in RT4 cells. Dot-like immunofluorescence was observed in both control and treated cells with a marked increase of immunofluorescence in RT4 cells treated with 50 μM fMLP for 6 h (Figures 3A,B) or with 1 μM CpG for 6 h (Figures 3C,D). Increased levels of phosphorylated paxillin in RT4 cells after treatment with 50 μM fMLP or 1 μM CpG for 6 h as well as decreased levels after FPR2 inhibition by PBP10 or inhibitory effect of INH ODN were demonstrated by western blot analysis (Figure 3E). These altered levels of phosphorylated paxillin corresponded to changes in numbers of cytoplasmic processes analyzed at the same conditions (Figures 1G,H).

### FPR2 and TLR9 Immunostaining Distal to Nerve Injury

**Figure 4:**
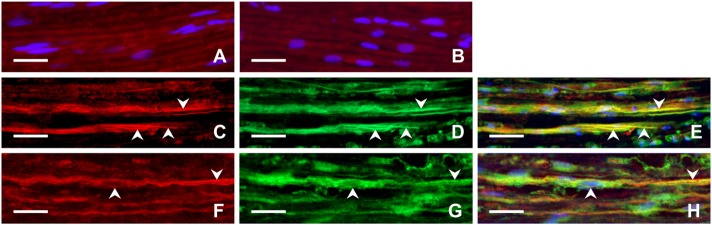
Representative longitudinal sections of the ulnar nerve removed from sham-operated rats **(A,B)** and distal to the ulnar nerve 3 days after transection **(C–H)**. The sections were immunostained with rabbit polyclonal anti-FPR2 **(A)** or anti-TLR9 **(B)** antibodies as well as double immunostained for FPR2 **(C)** or TLR9 **(F)** and then with chicken polyclonal anti-GFAP antibody **(D,G)**. Immunostaining was visualized with TRITC-conjugated donkey anti-rabbit **(A,B,C,F)** and FITC-conjugated anti-chicken secondary antibodies **(D,G)**. Merged pictures showed that GFAP+ activated Schwann cells and their cytoplasmic processes (arrowheads) displayed immunostaining for FPR2 **(E)** or TLR9 **(H)**. Cell nuclei were stained with Hoechst 33342. Scale bars = 40 μm.

**Figure 5:**
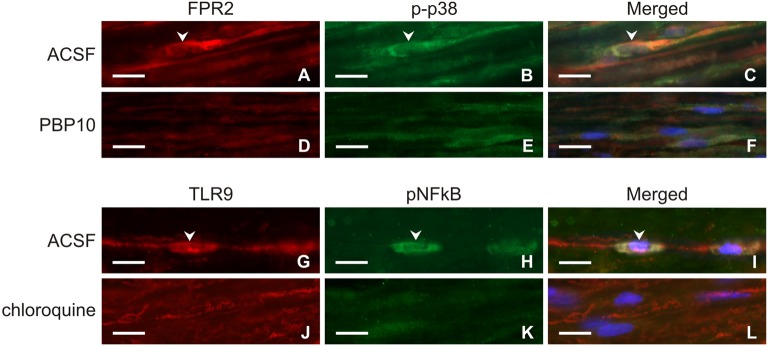
Representative longitudinal sections of the ulnar nerve distal to nerve crush after 3 days and intrathecal injection of artificial cerebrospinal fluid (ACSF; **A–C**, **G–I**), 1 μM PBP10 **(D–F)** or 50 μM chloroquine **(J–L)**. The sections were double immunostained for FPR2 **(A,D)** and p-p38 **(B,E)** or TLR9 **(G,J)** and pNFkB **(H,K)** under the same condition. Merged pictures revealed co-localization of p-p38 immunostaining in FPR2 immunopositive **(C)** and pNFkB immunofluorescence in TLR9 immunopositive **(I)** Schwann-like cells of rats with ACSF administration (arrowheads). Intrathecal injection of PBP10 or chloroquine significantly reduced FPR2/p-p38 **(D–F)** and TLR9/pNFkB **(J–L)** double immunostaining, respectively. Cell nuclei were stained with Hoechst 33342. Scale bars = 40 μm.

Longitudinal cryostat sections through the ulnar nerves from sham-operated rats displayed very low intensity of FPR2 and TLR9 immunofluorescence (Figures 4A,B). In contrast, increased intensity of TLR9 and FPR2 immunostaining was detected in sections cut distal to the nerve transection (Figures 4C,F). FPR2 or TLR9 immunopositivity was present in spindle-shaped cells and their processes that also displayed GFAP immunostaining indicating their Schwann cell origin (Figures 4D,E,G,H). Double immunodetection showed p-p38 MAPK immunostaining in FPR2 immunopositive Schwann-like cells distal to the ulnar nerve crush and intrathecal administration of ACSF. In contrast, intrathecal application of PBP10, an inhibitor of FPR2, reduced markedly immunofluorescence for both FPR2 and p-p38 MAPK (Figures 5A–F). Double immunostaining also revealed perinuclear pNFkB(p65) immunofluorescence in TLR9 immunopositive Schwann-like cells after ACSF administration, while the nerve sections from rats treated with chloroquine displayed significantly decreased intensity of both TLR9 and NFkB immunofluorescence (Figures 5G–L).

**Figure 6:**
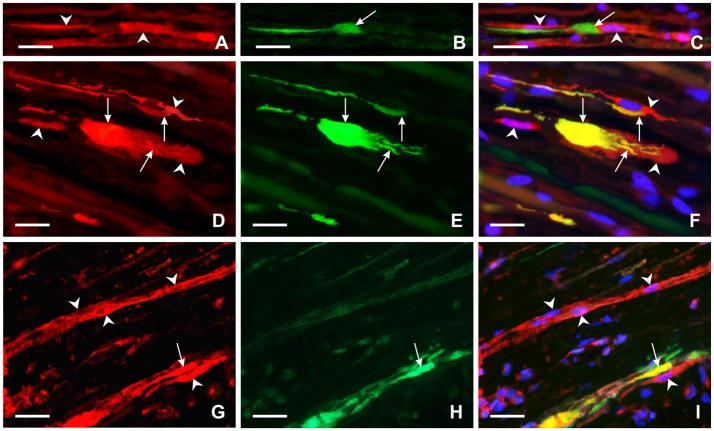
Representative longitudinal sections of the ulnar nerve distal to nerve crush after 3 days. Longitudinal section of the distal ulnar segment was double immunostained with chicken polyclonal anti-GFAP **(A)** and mouse monoclonal anti-GAP43 **(B)** antibodies. Immunoreaction was visualized with TRITC-conjugated anti-chicken **(A)** and FITC-conjugated anti-mouse secondary antibodies **(B)**. Cell nuclei were stained with Hoechst 33342. GFAP immunostained Schwann cells and their cytoplasmic processes (arrowheads) **(A,C)** were located close to the GAP43 immunopositive growth cone (arrow) **(B,C)**. Other longitudinal sections were double immunostained with rabbit polyclonal anti-FPR2 **(D)** or anti-TLR9 **(G)** antibodies and mouse monoclonal anti-GAP43 antibody **(E,H)**. Immunoreaction visualized with TRITC-conjugated donkey anti-rabbit **(D,G)** and FITC-conjugated donkey anti-mouse **(E,H)** secondary antibodies revealed that both FPR2 and TLR9 are present not only in cells close to (arrowheads) but also in growth cones and their processes (arrows) as shown in merged pictures **(F,I)**. Cell nuclei were stained with Hoechst 33342. Scale bars = 40 μm.

Activated GFAP-immunopositive Schwann cells were found close to regenerated axons and their growth cones distal to ulnar nerve crush (Figures 6A–C). Moreover, Schwann-like cells with FPR2 or TLR9 immunopositivity were frequently observed near the regenerated axons and their growth cones as visualized by GAP43 immunostaining. Primarily, we could demonstrate increased FPR2 and TLR9 expression in activated Schwann cells and their cytoplasmic process close to regenerated axons. Surprisingly, FPR2 or TLR9 immunostaining was also found in regenerated axons and mainly in their growth cones including filopodia (Figures 6D–I).

## Discussion

Schwann cells play a critical role in axon regeneration following peripheral nerve injury. Nerve injury-induced activation of Schwann cells is associated with extension of their cytoplasmic processes that promote axon growth and guidance of regenerated axons (Son and Thompson, 1995b; Gomez-Sanchez et al., 2017). Moreover, elaboration of cytoplasmic processes by Schwann cells is the main prerequisite for their migration to form a bridge spanning the gap between the distal and proximal stumps of severed nerves (Deumens et al., 2010) or other types of peripheral nerve lesions that then leads to nerve regeneration (Dubový and Svízenská, 1994; Liu et al., 2016).

Efficient axonal degeneration is essential for subsequent regeneration and functional recovery after nerve damage (Scheib and Höeke, 2013). Axonal degeneration of injured peripheral nerves involves granular degeneration of most axonal structures including mitochondria (Coleman, 2005; Park et al., 2013). Peripheral nerve axons contain a huge number of mitochondria which are a potent source of mtDAMPs as a result of their disintegration (Krysko et al., 2011). These include formylated peptides and mtDNA that can induce innate and inflammatory reactions after tissue damage (Zhang et al., 2010; Monlun et al., 2017).

fMLP and CpG were chosen as prototypic mtDAMPs for analyzing their effect on cytoplasmic process formation in RT4 schwannoma cells cultivated *in vitro*. Treatment with fMLP or CpG induced quantitative changes in cytoplasmic process extension in RT4 cells. The results indicated that formylated peptides and mtDNA released from mitochondria are involved, at the very least, in the initiation of cytoplasmic process extension by Schwann cells distal to nerve injury. This is in line with published evidence that mtDNA released by degenerating motor terminals can activate terminal Schwann cells and stimulate reinnervation of the neuromuscular junction through the MAPK pathway (Duregotti et al., 2015). In addition to mtDNA, terminal Schwann cells of degenerated motor terminals can be also activated by H_2_O_2_ triggering specific RNAs (Negro et al., 2018).

Our *in vitro* results revealed differences in the elaboration of cytoplasmic processes by various concentrations and durations of fMLP treatment. A higher concentration of fMLP (50 μM) was required to increase the number of cytoplasmic processes with a 1 and 6 h treatment, while a lower concentration (100 nM) was enough with longer exposure (24 h). In contrast, extended (24 h) exposure to high concentrations of fMLP (10 μM and 50 μM) had an inhibitory effect on elaboration of RT4 cell processes. These results suggest that a higher concentration of formylated peptides is needed for early initiation of cytoplasmic processes, but low concentration is enough to stimulate the formation of Schwann cell processes during prolonged exposure. The sort of fMLP dynamics revealed *in vitro* affecting the formation of cytoplasmic processes, may be close to the *in vivo* situation during activation of Schwann cells by formylated peptides during WD.

In contrast to fMLP, 1 μM CpG ODN induced a greater elaboration of cytoplasmic processes after 6 and 24 h treatment, while a shorter exposure for 1 h had no effect on the outgrowth of cytoplasmic processes in RT4 cells. Surprisingly, the inhibitory effect of INH ODN pre-treatment was found only after subsequent exposure with CpG ODN for 6 h, but 1 h and 24 h CpG ODN treatment following INH ODN pre-treatment had a synergistic effect on the formation of cytoplasmic extensions of RT4 cells. This synergistic effect of INH ODN pre-treatment on cytoplasmic extensions cannot yet be fully explained and further experiments are indicated.

Paxillin is an approximately 68 kDa cytoskeletal adaptor protein required for cell adhesion and outgrowth of cytoplasmic processes related to cell migration (Vindis et al., 2004; Achuthan et al., 2006; Romanova and Mushinski, 2011; López-Colomé et al., 2017). We detected significantly increased levels of phosphorylated paxillin in RT4 cells after treatment with 50 μM fMLP and 1 μM CpG ODN for 6 h compared to control cells. In addition, phosphorylated paxillin protein levels were reduced by preincubation of RT4 cells with PBP10 or INH ODN in experimental sets for 6 h corresponding with inhibitory effects on cytoplasmic process development. Our immunohistochemistry and western blot results revealed that both fMLP and CpG treatments for 6 h lead to increased phosphorylated paxillin levels concomitantly with increased formation of cytoplasmic processes. This is in agreement with enhanced phosphorylation of paxillin associated with cytoplasmic processes induced by fMLP or CpG in other cell types (Leventhal et al., 1997; Weiner et al., 2001; VanCompernolle et al., 2003; Vindis et al., 2004; Achuthan et al., 2006; Miyamoto et al., 2012). Moreover, fMLP is a known chemoattractant stimulating cell movement involving cytoplasmic processes (Xu et al., 2003).

Mitochondrial DAMPs produced by disintegrated mitochondria can be recognized by pattern-recognition receptors (PRRs) that include TLRs, FPR2, NOD-like receptors (NLRs), RIG-I-like receptors (RLRs) and purinergic receptors (Walsh et al., 2013; Wenceslau et al., 2013). Our results show that increased immunostaining for FPR2 and TLR9 was induced in RT4 cells treated with fMLP or CpG as prototypical ligands of these receptors, respectively. In addition, activated Schwann cells and their processes distal to nerve injury also displayed increased levels of both FPR2 and TLR9 indicating that these peripheral glial cells can react to mtDAMPs such as formylated peptides and mtDNA released from disintegrated axonal mitochondria during WD. Schwann cells and their processes positive for FPR2 and TLR9 immunostaining were frequently found close to growth cones of regenerated axons after nerve crush. Surprisingly, a distinct immunoreaction for these receptors was also observed in the growth cones indicating that these critical structures of regenerated axons can also react to formylated peptides and mtDNA present distal to nerve injury. The immunodetection of FPR2 and TLR9 in RT4 cells, activated Schwann cells and axonal growth cones indicates the involvement of both mtDAMP receptors in the overall mechanism of cell process formation.

It was demonstrated that FPR2 and TLR9 signaling pathway in glial cells contains activation of p38 MAPK and NFkB, respectively (Cattaneo et al., 2010; Lacagnina et al., 2018). To determine signaling events associated with FPR2 and TLR9 expression in activated Schwann cells after nerve injury, we used double immunostaining of these receptors with p-p38 MAPK and pNFkB(p65) in nerve segment distal to nerve crush after intrathecal administration of vehiculum (ACSF) in comparison to PBP10 or chloroquine as FPR2 and TLR9 inhibitors, respectively (Takeshita et al., 2004; Ho et al., 2018).

Our first observation of FPR2 localization in growth cones—thus, suggesting involvement of FPR2 signaling in axonal growth following nerve injury—is confirmed by the reduction of axon and dendrite outgrowth in developing hippocampal neurons after *in vitro* FPR2 inhibition (Ho et al., 2018). Moreover, FPR2 activation can promote axonal growth through F-actin polymerization—an important component of growth cones (Pacheco and Gallo, 2016; Wang et al., 2016). Besides formylated peptides, activation of FPR2 in growth cones may also be mediated by lipoxin A4 (LXA4) produced by myelin degradation during WD (Edström et al., 1996; De et al., 2003). For example, LXA4 binding to FPR2 can induce axonal or dendritic outgrowth as was revealed by treating primary hippocampal neurons with the FPR2 inhibitor PBP10, resulting in reduced axon and dendrite lengths (Ho et al., 2018). A role for TLR9 activation in the regenerating axon is suggested by a significant increase in both mRNA and protein levels of TLR9 in the developing mouse brain (Kaul et al., 2012). In contrast to FPR2, direct evidence for this TLR9 function is not yet available.

The *in vitro* results revealed a potentiated effect of INH ODN pre-treatment on the increased outgrowth of cytoplasmic processes of RT4 cells mainly following a 24 h CpG ODN treatment. Surprisingly, an inhibitory effect of INH ODN pre-treatment was observed on the extension of cytoplasmic processes at both concentrations used (1 μM, 10 μM), but only after CpG ODN stimulation of RT4 cells for 6 h. INH ODNs (e.g., TCCTGGCGGGGAAGT) selectively interfere with TLR9-mediated immunoactivation by competing with CpG ODN for binding to the C-terminal region of TLR9 (Avalos and Ploegh, 2011). Our results of the different efficacy of INH ODN pre-treatment on the induction of cytoplasmic processes in RT4 cells following various CpG ODN treatment times may reflect varying competition between INH ODN and CpG ODN.

## Conclusion

We proved that fMLP and CpG ODN act as prototypic mtDAMPs and modulate the outgrowth of cytoplasmic processes of RT4 schwannoma cells in association with increased levels of phosphorylated paxillin. fMLP and CpG ODN affected formation of RT4 cell cytoplasmic processes in a dose- and time-dependent fashion. RT4 cells displayed increased immunostaining for FPR2 and TLR9 following treatments with fMLP or CpG, the ligands of these receptors, respectively. FPR2 and TLR9 immunofluorescence staining was also found in Schwann cells and their processes distal to nerve injury and close to growth cones suggesting a possible activation of the glial cells by mtDAMPs released after nerve injury. In addition to Schwann cells, both FPR2 and TLR9 were observed also in growth cones suggesting their involvement in axon regeneration. The results indicate the involvement of both FPR2 and TLR9 activation in the general mechanisms of cell process formation.

## Author Contributions

AK designed the experiments, ensured *in vitro* cells, conducted western blot analyses, participated in acquiring and analyzing the presented data and wrote the manuscript. IK, IH-S and MJ designed and performed the *in vivo* experiments. PD conceived, designed, coordinated the experiments and wrote the manuscript. All authors have approved the final version for publication.

## Conflict of Interest Statement

The authors declare that the research was conducted in the absence of any commercial or financial relationships that could be construed as a potential conflict of interest.
